# P-1287. Incidence of Symptomatic Lyme Borreliosis in Nine European Countries, 2018−2022

**DOI:** 10.1093/ofid/ofae631.1468

**Published:** 2025-01-29

**Authors:** Frederick Angulo, Emily Colby, Volker Fingerle, Anne-Mette Lebech, Per-Eric Lindgren, Anna Moniuszko-Malinowska, Franc Strle, Julia Olsen, Gordon Brestrich, Andrew Vyse, Madiha Shafquat, L Hannah Gould, Patrick Kelly, Andreas Pilz, Kate Halsby, Jennifer Moisi, James H Stark

**Affiliations:** Pfizer Vaccines, Portland, Oregon; Pfizer Vaccines, Portland, Oregon; Ludwig-Maximilians University, Munich, Bayern, Germany; University Hospital Copenhagen-Rigshospitalet, Copenhagen, Nordjylland, Denmark; Linköping University, Linköping, Ostergotlands Lan, Sweden; Medical University of Bialystok, Bialystok, Podlaskie, Poland; University of Ljubljana, Ljubljana, Ljubljana, Slovenia; Pfizer Vaccines, Portland, Oregon; Pfizer Vaccines, Portland, Oregon; Pfizer UK, Tadworth, England, United Kingdom; Pfizer Vaccines, Portland, Oregon; Pfizer Vaccines, Portland, Oregon; Pfizer Vaccines, Portland, Oregon; Pfizer Corporation Austria, Vienna, Wien, Austria; Pfizer Inc, London, England, United Kingdom; Pfizer Vaccines, Portland, Oregon; Pfizer Biopharma Group, Collegeville, Pennsylvania

## Abstract

**Background:**

Lyme borreliosis (LB) is the most common tick-borne disease in Europe. Many European countries conduct LB surveillance. However, many symptomatic Bbsl infections are not detected by surveillance. The aim of this study was to estimate the incidence of symptomatic *Borrelia burgdorferi* sensu lato (Bbsl) infection, including cases not detected or reported by LB surveillance.

**Figure:**
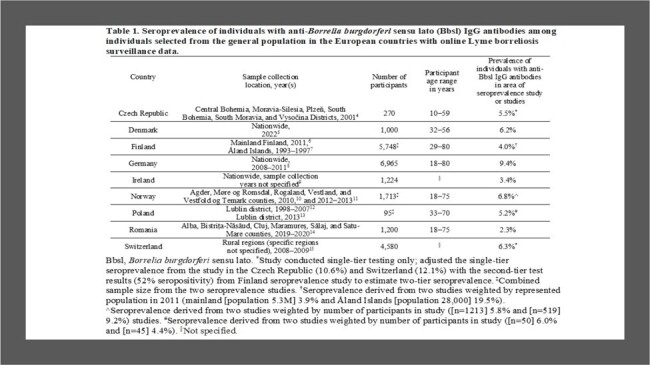

**Methods:**

Seroprevalence studies of anti-Bbsl antibodies published in 1998−2023, along with estimates of the symptomatic proportion and persistence of antibodies among Bbsl-infected individuals from studies published in 1990−2023, were used to estimate the incidence of symptomatic Bbsl infection. This incidence was then compared to the incidence of surveillance-reported LB to derive country-specific multipliers and estimate the incidence of symptomatic Bbsl infection from 2018−2022.

**Figure:**
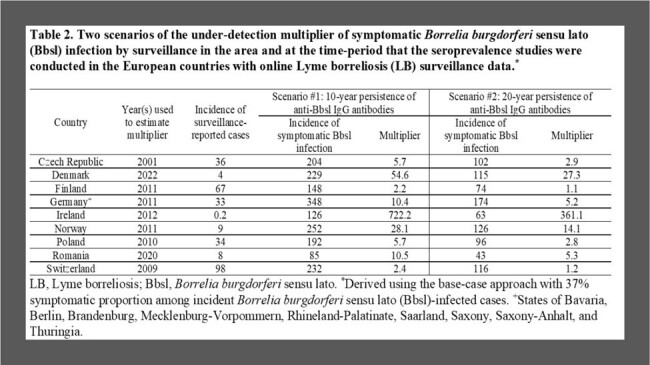

**Results:**

Seroprevalence and LB surveillance data were available in nine countries; the prevalence of individuals with anti-Bbsl IgG antibodies ranged from 2.3% in Romania to 9.4% in Germany (Table 1). The pooled estimate of the symptomatic proportion among Bbsl-infected persons from five studies was 37% (95% confidence interval 28%­­−46%). There was a wide range (0.4−33.3 years) of persistence of antibodies in seven studies; therefore, a 10-year duration (scenario #1) and 20-year duration (scenario #2) were used for the duration of antibody detection. Under-detection multipliers varied across surveillance systems (Table 2). Under scenario #1, under-detection multipliers were 2.3−10.5 in countries reporting all LB cases and 54.6−772.2 in countries reporting only Lyme neuroborreliosis (LNB) cases. Under scenario #2, under-detection multipliers were 50% lower than the multipliers under scenario #1. Under both scenarios, the incidence of symptomatic Bbsl infection adjusted for under-detection was highest in Finland, Germany, Norway, Poland, and Switzerland, intermediate in the Czech Republic and Denmark, and lowest in Ireland and Romania (Table 3).

**Figure:**
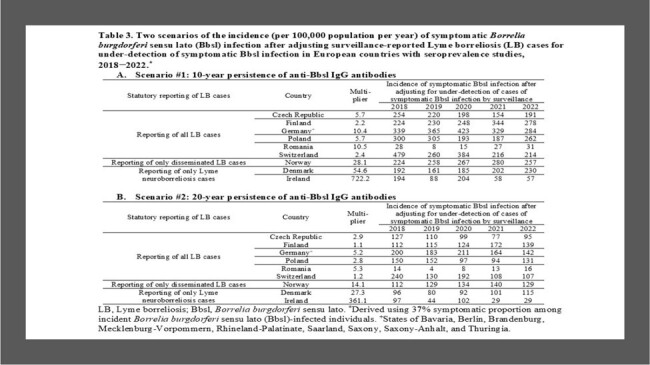

**Conclusion:**

After adjustment of LB surveillance data for under-detection, we found a high incidence of symptomatic Bbsl infection in several European countries. Differences in surveillance need to be considered when comparing reported data across countries.

**Disclosures:**

**Frederick Angulo, DVM PhD**, Pfizer Vaccines: Employee|Pfizer Vaccines: Stocks/Bonds (Public Company) **Gordon Brestrich, PhD**, Pfizer Vaccines: Employee|Pfizer Vaccines: Stocks/Bonds (Public Company) **Andrew Vyse, Ph.D.**, Pfizer Vaccines: Employee|Pfizer Vaccines: Stocks/Bonds (Public Company) **L. Hannah Gould, PhD, MS, MBA**, Pfizer: Employee|Pfizer: Stocks/Bonds (Public Company) **Patrick Kelly, PhD**, Pfizer Vaccines: Employee|Pfizer Vaccines: Stocks/Bonds (Public Company) **Andreas Pilz, PhD**, Pfizer Vaccine: Stocks/Bonds (Public Company) **Kate Halsby, PhD**, Pfizer Vaccines: Stocks/Bonds (Public Company) **Jennifer Moisi, PhD**, Pfizer: Employee|Pfizer: Stocks/Bonds (Public Company) **James H. Stark, PhD**, Pfizer: Employee|Pfizer: Stocks/Bonds (Public Company)

